# Comparison of volumetric and areal bone mineral density in CT and scout scans using spectral detector technology

**DOI:** 10.1186/s41747-023-00356-7

**Published:** 2023-08-01

**Authors:** Johannes Hammel, Lorenz Birnbacher, Graeme Campbell, Philippe Coulon, Lev Ushakov, Franz Pfeiffer, Marcus R. Makowski, Jan Kirschke, Daniela Pfeiffer

**Affiliations:** 1 Department of Diagnostic and Interventional Radiology, TUM School of Medicine, Klinikum Rechts Der Isar, Technical University of Munich, Munich, Germany; 2 Chair of Biomedical Physics, Department of Physics, TUM School of Natural Sciences, Technical University of Munich, Garching, Germany; 3 Philips GmbH Market DACH, Hamburg, Germany; 4 Philips Healthcare, Suresnes, France; 5 Munich Institute of Biomedical Engineering, Technical University of Munich, Garching, Germany; 6 TUM Institute for Advanced Study, Technical University of Munich, Garching, Germany; 7 Department of Diagnostic and Interventional Neuroradiology, TUM School of Medicine, Klinikum Rechts Der Isar, Technical University of Munich, Munich, Germany

**Keywords:** Musculoskeletal diseases, Bone density, Tomography (x-ray computed), Osteoporosis, Absorptiometry (photon)

## Abstract

**Background:**

To determine whether denoised areal bone mineral density (BMD) measurements from scout scans in spectral detector computed tomography (CT) correlate with volumetric trabecular BMD for opportunistic osteoporosis screening.

**Methods:**

A 64-slice single-source dual-layer spectral CT scanner was used to acquire scout scan data of 228 lumbar vertebral bodies within 57 patients. Scout scans in anterior–posterior (AP) view were performed with a dose of < 0.06 mSv and spectrally decomposed into areal BMD (aBMD) values. A spectral dictionary denoising algorithm was applied to increase the signal-to-noise ratio (SNR). Volumetric trabecular bone mineral density (vBMD) was determined via material decomposition. A 3D convolutional network for image segmentation and labeling was applied for automated vBMD quantification. Projected maps were used to compare the classification accuracy of AP and lateral scout scans.

**Results:**

The denoising algorithm led to the minimization of anticorrelated noise in spectral maps and an SNR increase from 5.23 to 13.4 (*p* < 0.002). Correlation analysis between vBMD and measured AP aBMD, projected AP, and lateral aBMD showed a Pearson correlation coefficient of 0.68, 0.81, and 0.90, respectively. The sensitivity and specificity for the osteoporosis classification task were higher in lateral projection images than in AP crystallizing in an increased area under the curve value of 0.99 *versus* 0.90.

**Conclusion:**

Denoised material-specific aBMD maps show a positive correlation to vBMD, enabling spectral scout scans as an opportunistic predictor for osteoporotic patients. This could be applied routinely as a screening tool in patients undergoing a CT examination.

**Relevance statement:**

Scout-based DEXA could be applied routinely as a screening tool in patients undergoing a CT examination.

**Key points:**

• Spectral scout scans can be used as a dual-energy x-ray absorptiometry-like screening tool.

• Spectral dictionary denoising on projection images increases the signal-to-noise ratio.

• Positive correlation between volumetric and areal bone mineral density is observed.

• Lateral projections increase osteoporosis classification accuracy compared to anterior-posterior projections.

**Graphical Abstract:**

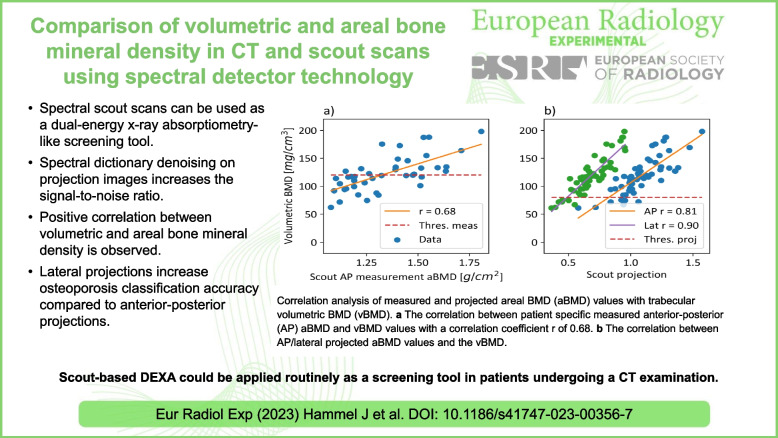

## Background

With an aging population worldwide, osteoporosis and resulting fragility fractures become a socio-economic burden resulting in an increasing need for early diagnosis and treatment [1]. Twenty-two million women and 5.5 million men were estimated to have osteoporosis [2] in the European Union, but only a minority of patients receive treatment [3, 4]. This phenomenon is called the treatment gap for osteoporosis. In the USA, after an osteoporotic fracture, only 9% of the patients underwent consecutive osteoporosis testing [1]. The World Health Organization reference standard for diagnosing osteoporosis is the dual-energy absorptiometry (DEXA) technique applied at the femur neck or lumbar spine [5]. The areal bone mineral density (aBMD) is determined by spectrally separated measurements using a K-edge filter and varying acceleration voltages. The marginal diagnostic rate may be a result of reduced sites with DEXA availability and therefore limited screening possibilities [6]. To overcome this treatment gap, along with the huge socioeconomic burden of fractures in older people, universal access to such facilities should be supported [7]. Other available x-ray-based techniques are the three-dimensional (3D) quantitative CT (QCT) volumetric BMD (vBMD) assessment on conventional CT scanners using a reference phantom. Promising results for the diagnosis with significantly increased osteoporosis detection rates from 17.1 to 46.4% for DEXA and QCT, respectively [8], were shown. With the introduction of spectral CT systems, a retrospective, opportunistic, phantom-less quantification of volumetric BMD was made possible by using virtual monoenergetic images for the material decomposition into bone and soft tissue maps [9–13]. The niche technology high-resolution peripheral QCT allows the assessment of complementary quality parameters for bone morphology or simulating loading conditions via finite element analysis [14]. Further methods for the assessment of bone health not relying on x-rays are quantitative ultrasound [15] and magnetic resonance imaging [16], *e.g.*, for quantification of trabecular structures [3, 4, 6, 7].

Our study explores an approach to automated BMD testing for radiology departments using spectral CT scout scans (also called overview or topogram) from a detector-based dual-energy system. Spectral scouts can be used for a projection-based material decomposition to opportunistically determine aBMD values in patients undergoing a CT examination, which is also known as scout-based dual-energy absorptiometry (SDEXA) [11, 12].

In principle, DEXA and SDEXA could use almost the same processing. DEXA needs spectral information, which is generated by filtration, while scout scans from a dual-energy CT system can use a spectral detector fulfilling this task. Both methods then need the calculation of the aBMD and segmentation. First, we show how to calculate aBMD data and present a denoising algorithm improving the SNR of aBMD results. Denoising is applied, as scout acquisitions are conducted with a very low dose and additionally material decomposition introduces anti-correlated noise onto the aBMD material maps. As a standard of reference, spectrally determined vBMD values from the subsequent CT measurement are used, allowing the generation of projected aBMD masks from CT data to obtain a matched comparison between vBMD and aBMD. With our approach using the same CT system, where we compare SDEXA with vBMD, we know the beam geometry and position. This allowed us to match vBMD with SDEXA in a more precise way. In comparison to Laugerette et al. [11, 12], where phantom measurements and fracture differentiation on scout measurements were analyzed, we investigated the correlation between measured aBMD and vBMD. Furthermore, we generated projected aBMD images in anterior–posterior (AP) and lateral views from the spectral CT data and compared the osteoporosis classification accuracy for both views.

The aim of this study was to assess the clinical performance of SDEXA measurements using volumetric BMD as a ground truth.

## Methods

### Study design and patient selection

This study is a retrospective secondary analysis of an unpublished study aiming to investigate the accuracy of spectral lateral scout scans for diagnosing osteoporosis. According to planned sample size calculations, 57 patients with an average age of 43 years from ages 17 to 80 and a sex distribution of 23 female and 34 male subjects were enrolled in that study. The patient collective included 47 AP and 10 lateral measurements. For the purposes of this secondary analysis, two patients were excluded because of the overlay of intravenous or oral contrast agents, and all lateral scout scan patients were excluded because of the limited image quality of the lateral scout scans resulting from not adapted dose levels. Contrast agent overlay can cause severe overestimation of aBMD values comparably to QCT [17] or the overlay of osteoarthritis in DEXA [18]. Furthermore, 10 patients with low-quality AP scout scans were excluded from the analysis as no dose adaptation was used for larger patients. All examinations were performed between March and September 2021, as the automated segmentation tool named “anduin” [19] was maintained and offline after that period. Further statistical verification with an increased patient population is in the pipeline.

### Protocol settings

A standard CT abdomen protocol with a fixed tube voltage of 120 kVp and an exposure of from 20 to 122 mAs per rotation (1.80 to 10.5 mGy CT dose index volume) was used on a 64-slice single source dual-layer CT scanner with a detector coverage of 4 cm and a rotation time of 0.33 s (IQon, Philips Healthcare, Best, The Netherlands). Scout scans were acquired with a peak tube voltage of 120 kVp and a tube current of 30 mA. The CT dose index volume and dose length product, estimated dose converted from two-dimensional (2D) to 3D were approximately 0.06 mGy and 3.5 mGy*cm (< 0.06 mSv, *k* = 0.015 for abdomen and pelvis) in scout measurements and on average 7.2 mGy and 400 mGy*cm (≈ 6.0 mSv, *k* = 0.015 for abdomen and pelvis) in abdomen CT protocols. Spectral raw data were reconstructed using a standard soft tissue filter kernel (type B) with an axial slice thickness of 0.9 mm. The isotropic pixel spacing in the *x*–*y*-plane ranged from 0.56 to 0.97 mm physical distance between the center of each pixel (generated with IntelliSpace Portal 11.0, Philips Healthcare, Best, The Netherlands).

### Denoising in material-selective images

Anticorrelated noise appears on material decomposed images with structural correlation [20]. An algorithm by Mechlem et al. [21] was adapted to reduce noise amplification in spectral material maps.

The anticorrelated noise contribution can be minimized by a weighted addition of spectral maps generating a reference image at a certain energy where anticorrelated noise maximally cancels out. We refer to this as the minimum noise image. Dictionary denoising separates image features from noise by using a sparse representation by natural image patches. These so-called dictionary atoms are linearly combined to fit the original noisy image. Dictionary denoising on the minimum noise image was applied for the identification of structures and edges. Denoised basis material images were calculated by applying a local linear transformation to the processed minimum noise image.

### BMD calculation

**Fig. 1:**
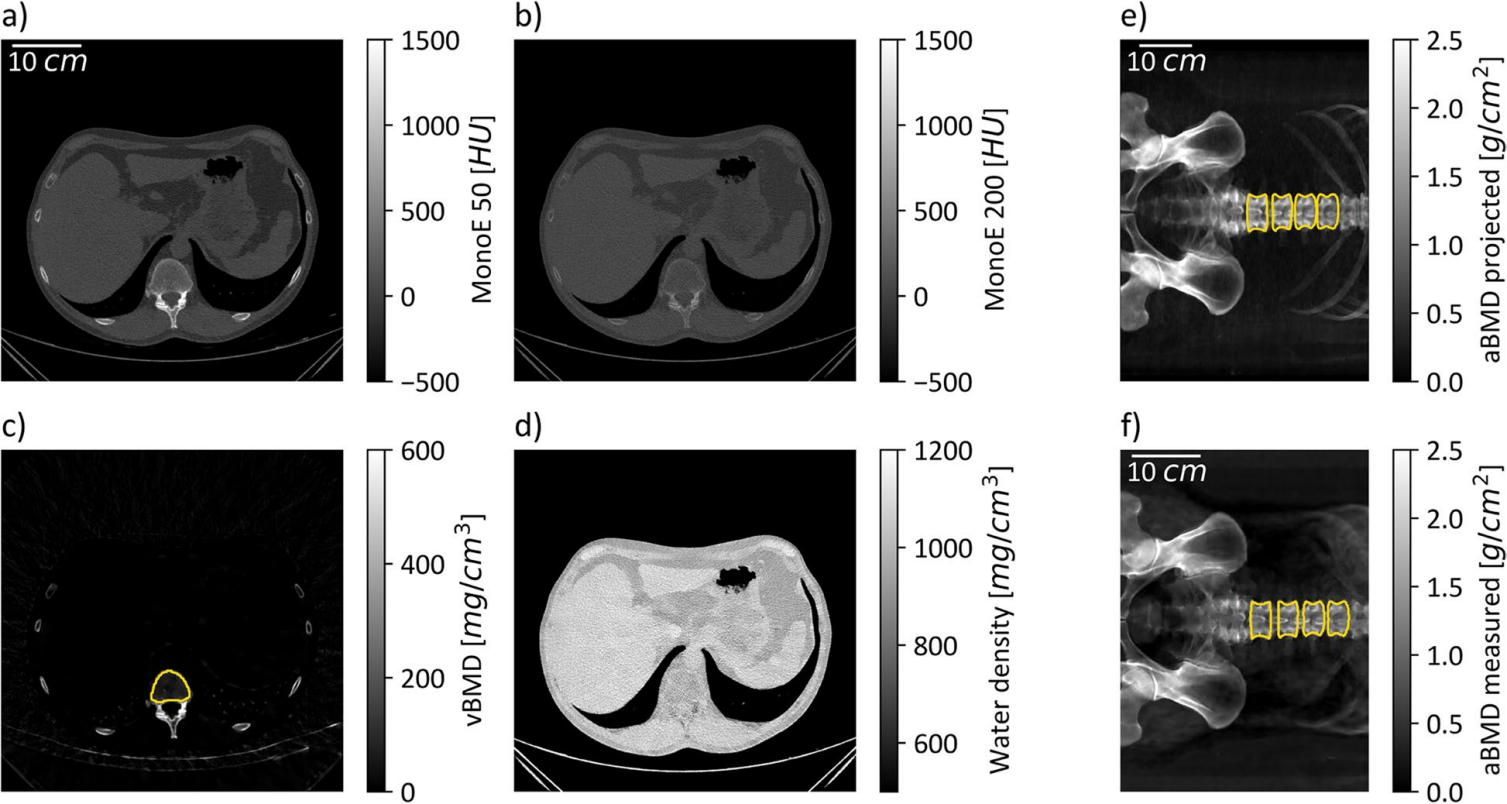
Schematic of bone mineral density (BMD) quantification in three-dimensional and two-dimensional data. **a**, **b** The monoenergetic images at 50 and 200 keV at the same windowing in a certain slice. By solving the linear equation system given in Eq. 1 in every voxel, volumetric BMD (vBMD) (**c**) and water maps (**d**) are calculated. The yellow mask in **c** shows the trabecular bone mask generated with the anduin tool. **e** The anterior–posterior projected areal BMD (aBMD) map and projected borders of the trabecular mask. **f** The aBMD map calculated directly from the spectral scout measurement overlayed with the borders of the projected and registered trabecular mask

**Fig. 2:**
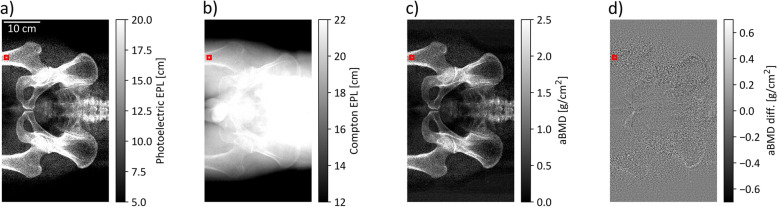
Overview of spectral maps. The photoelectric (**a**) and Compton (**b**) water equivalent path length (EPL) images are combined to generate an areal BMD (aBMD) map (**c**) in every patient. The aBMD maps can be generated from raw photoelectric and Compton images, or by using the denoised EPL data. **d** The different image of the aBMD map generated from raw and denoised spectral maps. An increased difference can be observed in bone regions like the hip or femur

Figure 1 gives a schematic overview of how measured and projected results were calculated. To generate aBMD (Figs. 1f and 2c) maps from spectral scout scans, a raw data extraction tool from the CT machine manufacturer was provided. Using the raw data files from the scanner, water equivalent path length (EPL), photoelectric and Compton images (Fig. 2a, b) and resultant virtual monoenergetic projection values can be determined (refer to electronic supplementary material in [12]). No soft tissue correction factor was applied as it resulted in an aBMD offset for patients with oral and intravenous contrast agents in the ROI selected for soft tissue correction. 2D masks corresponding to aBMD images for the quantification of vertebra-specific aBMD values are generated automatically. For this, the freely available bonescreen anduin research tool [19] was used to generate a labeled CT segmentation (Fig. 1c, yellow mask) of all vertebral bodies within the scout-associated CT measurement.

The labeled vertebral bodies were distinguished in trabecular and cortical voxels. The trabecular mask was used as an input to the forward projection algorithm, which created projected scout scans from CT images (Fig. 1e) [22]. The forward projection algorithm was implemented using the appropriate CT geometry parameters and a banana-shaped detector focused on the focal spot of the x-ray tube. A fan angle of 52.5° covers 512 detector columns. In *z*-direction, a scanning approach was simulated, where the patient is moved through a collimated beam with a coverage of four detector pixels in *z*-direction.

By adjusting the projection angle to 0 or 90°, AP and lateral scout images could be projected. Volumetric BMD images (Fig. 1c), as well as 3D spine masks (Fig. 1c, yellow mask), served as input for the projection algorithm. By projecting the vBMD maps, artificially generated aBMD maps in lateral and AP view were generated for every patient. Correlation and classification analysis was performed on those projected aBMD maps, as well as measured aBMD maps from spectral scout images. To obtain a trabecular-based 2D mask for aBMD quantification on scout measurements (Fig. 1f, yellow mask), projected aBMD maps (Fig. 1e) together with an image registration step were used. The open-source software SimpleITK [23, 24] served as an affine and translatory registration library.

An aBMD value for L1 to L4 was obtained in all patients. The volume of interest to calculate a spectral vBMD was automatically segmented using the trabecular CT masks obtained from the bonescreen anduin research tool (Fig. 1c, yellow mask); vBMD maps were generated by a material decomposition from virtual monoenergetic images (Fig. 1a, b) into hydroxyapatite and water maps (Fig. 1c, d) via the solution of the following linear equation system:

$$\left(\begin{array}{c}\begin{array}{c}{\mu }_{50}\\ {\mu }_{200}\end{array}\end{array}\right)=A\cdot \left(\begin{array}{c}{\rho }_{water}\\ {\rho }_{bone}\end{array}\right)=\left(\begin{array}{cc}{\left(\frac{\upmu }{\uprho }\right)}_{water}\left(50\right)& {\left(\frac{\upmu }{\uprho }\right)}_{bone}\left(50\right)\\ {\left(\frac{\upmu }{\uprho }\right)}_{water}\left(200\right)& {\left(\frac{\upmu }{\uprho }\right)}_{bone}\left(200\right)\end{array}\right)\cdot \left(\begin{array}{c}{\rho }_{water}\\ {\rho }_{bone}\end{array}\right),$$

where ${\mu }_{50/200}$ are the attenuation coefficients at monoenergetic images 50 and 200 keV, ${\left(\frac{\mu }{\rho }\right)}_{\mathrm{water}/\mathrm{bone}}$ (50/200) are the mass attenuation coefficients of water and bone at 50 and 200 keV, and ${\rho }_{\mathrm{water}/\mathrm{bone}}$ are the hydroxyapatite and water density maps.

Three different approaches for measuring the patient-specific aBMD value were compared to the spectrally assessed trabecular vBMD (Fig. 4):

- Scout-based DEXA (SDEXA) using denoised spectral scout measurements along with material decomposition into bone (aBMD) and water images (Fig. 4a);
- AP projected aBMD from vBMD maps equivalent to measured aBMD (Fig. 4b);
- lateral aBMD projections preventing the overlay of trabecular structures with the spinal process (Fig. 4c, e).

### Statistical analysis

The open-source scientific computing library for Python SciPy [25] was used to calculate a linear least-squares regression to correlate the 3D with 2D BMD measurements. The slope, intercept, Pearson correlation coefficient, and *p*-value for a hypothesis test whose null hypothesis is that the slope is zero were obtained. Furthermore, the standard error of the estimated slope was assessed, and a 95% confidence interval on slope and intercept was calculated by using a two-sided inverse Student *t*-distribution. In addition, a two-sided *t*-test for the null hypothesis that two independent samples have identical mean values was used. To compare the classification accuracy of aBMD measurements, a receiver operating characteristic (ROC) for a binary classification task was consulted [26]. The area under the curve (AUC) was calculated by a general function for integration using the trapezoidal rule.

## Results

### Denoising in material selective images

**Fig. 3:**
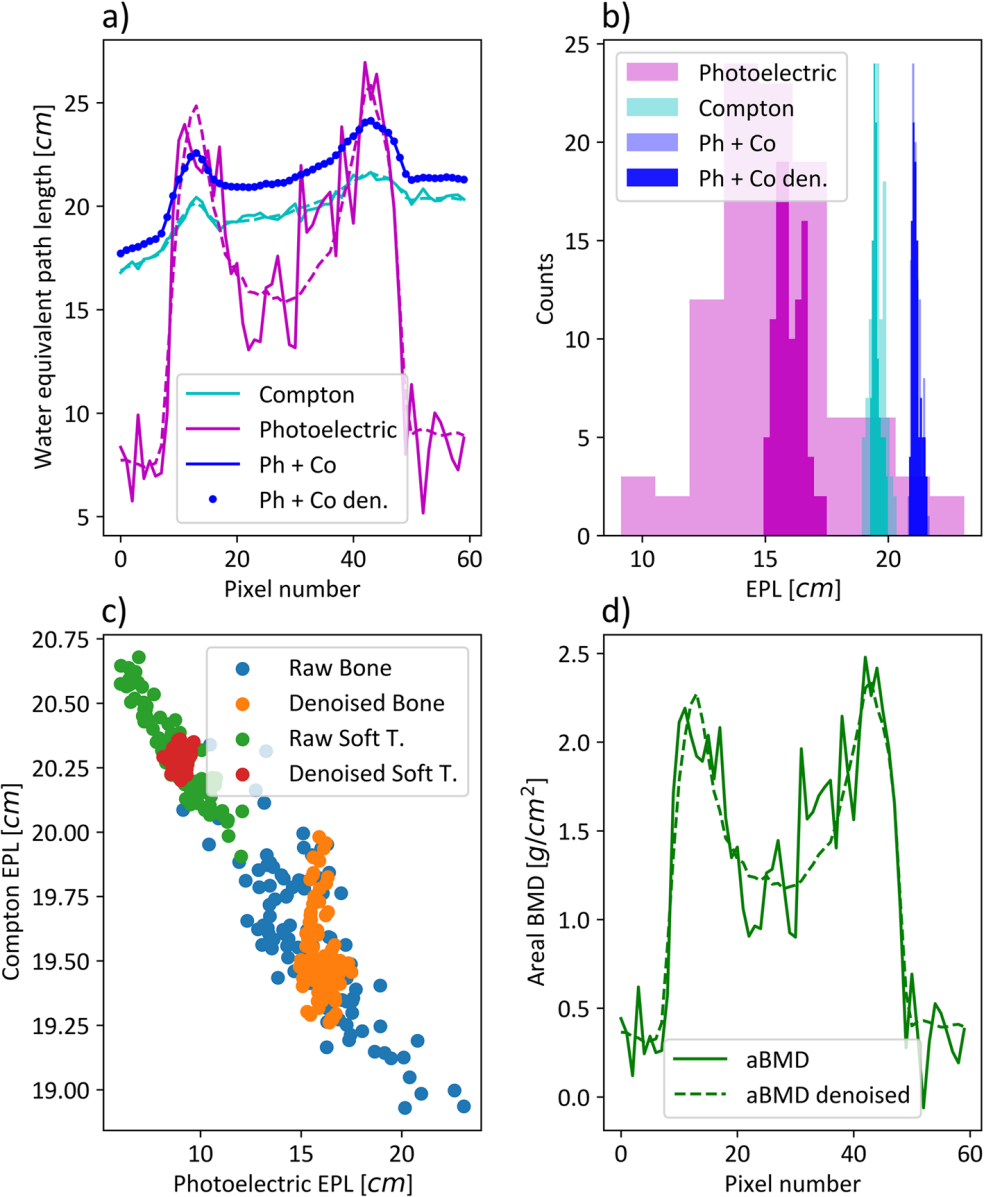
Behavior of anticorrelated noise and spectral denoising. **a** The line profile through the femur bone in a representative patient. The raw data is plotted using a solid linestyle, while denoised line profiles are plotted with dashed lines or dotted in the case of weighted addition (Ph + Co). **b** A histogram of photoelectric, Compton and weighted combination for raw (translucent) and denoised (opaque) bone data in a ROI (see Fig. 2, red square). **c** A scatterplot of raw and denoised datapoints in a soft tissue and bone ROI. The anticorrelation of spectral maps and the reduction of this behavior by denoising can be demonstrated. **d** The line profile equivalent like in **a** for areal BMD (aBMD) maps generated from spectral data. *EPL*, Equivalent path length; *Ph* + *Co*, Photoelectric plus Compton; *Ph* + *Co den.*, Photoelectric plus Compton denoised; *Soft T.*, Soft tissue

The signal-to-noise ratio (SNR) in a homogenous soft tissue region of interest (ROI) without bone contribution could be significantly increased for photoelectric images (*p* < 0.002) from a mean value of 5.23 to 13.4. No significant increase could be observed in the equivalent ROI in Compton images (*p* = 0.708 > 0.05). The noise reduction algorithm shows qualitatively superior performance in regions with bone contribution (Fig. 2d), which is more problematic to quantify, as homogenous bone regions are hard to obtain in scout images. In an ROI of a patient’s femur (Fig. 2, red squares), an SNR increase from 6.16 to 28.7 was determined in photoelectric EPL images and 3.93 to 18.7 in aBMD images. A line plot through the red square region was assessed in Fig. 3a. The two peaks, especially visible in the photoelectric EPL line, illustrate the cortical bone, while the dip in between corresponds to the cancellous bone. The dashed and solid lines show the line profile along the denoised and raw datasets in photoelectric and Compton EPL images. The solid blue line visualized the profile along the weighted addition or minimum noise image. It is important that the weighted addition of raw spectral images and denoised datasets are alike. This is shown by the equal course of the solid and dotted blue lines. Figure 3b shows a histogram obtained for pixel values from the red square ROI in Fig. 2, illustrating the noise suppression of the denoising algorithm. The translucent histograms correspond to raw data, while the dense histograms show denoised data. The EPL changes for Compton and combined datasets are small, indicated by the result of no significant increase in the SNR. The standard deviation in photoelectric images differs strongly. Figure 3c indicates the anticorrelation characteristic of spectral results. The Pearson correlation coefficient in the bone and soft tissue ROIs equal -0.80 and -0.93. The anticorrelation was reduced to -0.22 and -0.008 in denoised spectral maps. Figure 3d, equivalent to 3a, plots the profile in the aBMD map for raw and denoised data. As the contribution of photoelectric EPL maps is much larger for aBMD calculation than the Compton contribution, the noise suppression due to the spectral denoising algorithm derivatives from the photoelectric EPL behavior in Fig. 3a. The denoising step alters the mean per patient measured aBMD densities only to a small extent, with a maximum per patient deviation of 1.7%.

### Correlation analysis

**Fig. 4:**
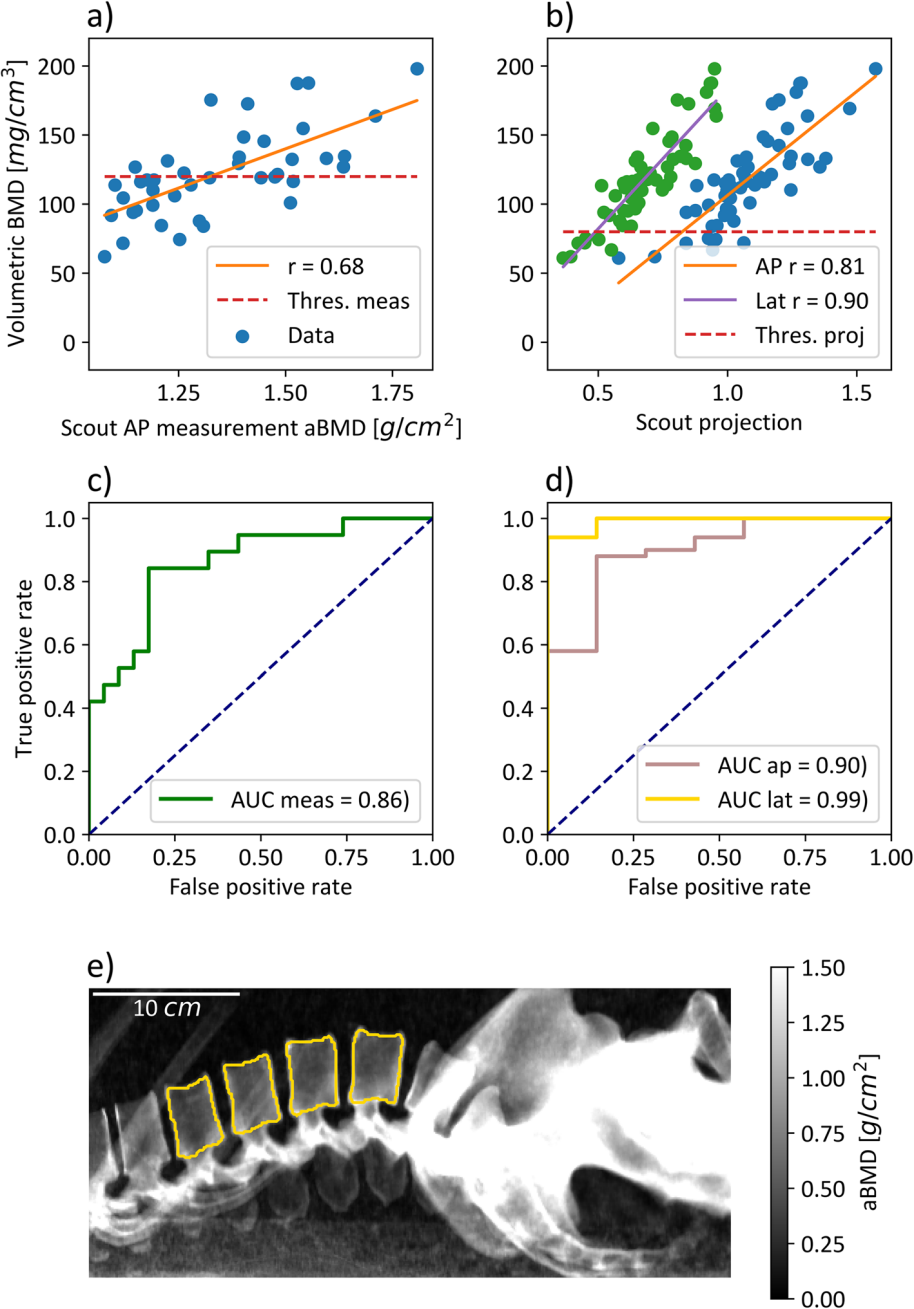
Correlation and classification analysis of measured and projected areal BMD (aBMD) values with trabecular volumetric BMD (vBMD). **a** The correlation between patient specific measured anterior–posterior (AP) aBMD and vBMD values with a correlation coefficient r of 0.68. **b** The correlation between AP/lateral projected aBMD values and the vBMD. At receiver operating characteristic analysis, the thresholds 120 and 80 mg/mL were used for measured and projected data, respectively. **c**, **d** The classification analysis on measured and projected aBMD values for correctly classifying osteoporotic patients based on the ground truth of trabecular vBMD values. **e** An example image of a lateral projected aBMD map. Note that one can separate between the trabecular and spinal process structures. Furthermore, an overlay of the rips is visible in lumbar vertebrae L1 and L2

The spectral trabecular vBMD served as a ground truth classification of the osteoporotic status in every patient, with a BMD < 80 mg/mL being classified as osteoporotic, leading to 6 females and 1 male out of 57 patients being classified as osteoporotic. This threshold was adapted from the American College of Radiology guidelines [27, 28]. The correlation coefficient between trabecular vBMD and measured AP aBMD, projected AP aBMD and projected lateral aBMD was found to be 0.68, 0.81, and 0.90, respectively. The calculated slopes and their 95% confidence interval were 113.6 ± 39.5, 150.4 ± 29.7, and 203.0 ± 27.2 in [1/cm]. The classification accuracy for projected data was compared on AP and lateral aBMD values (Fig. 4d) using the AUC value. The classification on lateral projected aBMD with the reference of spectral trabecular vBMD led to an AUC of 0.99 with a true positive rate of 94% and a false positive rate of 0% using the threshold 0.57 g/cm^2^. These values were selected based on the minimum distance to 100% true positive rate and 0% false positive rate. For detailed values on the true positive rate and false positive rate, refer to Table 1.

**Table 1:**
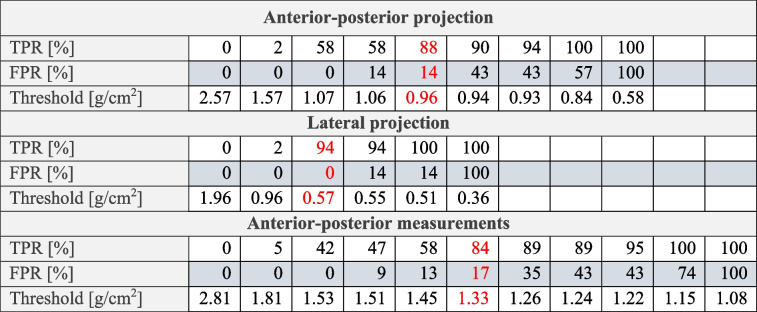
True positive rate (TPR), false positive rate (FPR), and threshold values of the analyzed receiver operating characteristic curves

Minimum distance values are highlighted in red

The classification accuracy using projected AP values decreased to an AUC of 0.90 with a true positive rate of 88% and a corresponding false positive rate of 14% using a threshold of 0.96 g/cm^2^. The equivalent ROC was not calculated for scout-derived AP aBMD values, as 4 of 7 osteoporotic individuals had to be excluded because of noisy scout images. Instead, the threshold BMD < 120 mg/mL was used as classifying osteopenic and osteoporotic patients *versus* normal, as the statistic in scout measurements was sufficient with 19 normal and 23 osteoporotic/osteopenic patients. An AUC of 0.86 with a true positive rate of 84% and a corresponding false positive rate of 17% using a threshold of 1.33 g/cm^2^ were measured (Fig. 4c). The denoising of measured data had no influence on the ROC.

## Discussion

DEXA is a 2D spectral technique for the assessment of aBMD and is the reference standard for diagnosing osteoporosis [29]. Several studies indicate that the volumetric assessment of BMD performs substantially better as a predictor for prevalent and consecutive vertebral fractures compared to DEXA [30–32], where lateral aBMD measurements outperform AP acquisition [33]. Nevertheless, the radiation dose of a high-quality quantitative CT protocol at the spine is much higher, *e.g.*, about 1.0 and 1.6 mSv for men and women assessed using Monte Carlo calculations, respectively [34].

The main results of our study were the findings of positive correlation between the 2D SDEXA aBMD with trabecular volumetric BMD from 3D spectral CT images and the reduction of anticorrelated noise in spectral scout maps by a dictionary-based denoising algorithm.

In fact, to avoid unnecessary exposure to patients from CT scans, spectral scout images could be used as a preliminary indicator for osteoporosis. Scout measurements are taken in every patient to select the field of view for the CT protocol and to apply automated dose modulation. By extending the scout protocol to the lumbar spine region in every patient, aBMD values could be acquired as additional pre-CT information, also in CT protocols where the lumbar spine is not in the field of view. In case of low aBMD values, the CT protocol could be extended for the examination of the lumbar spine region to perform a follow-up diagnosis of the osteoporosis status. Further studies should focus on how well the SDEXA technique can detect consecutive fractures. For this approach, reasonable image quality must be achieved in scout measurements. Material decomposition algorithms on spectral image data lead to low SNR in material selective images [35].

In our work, to decrease image noise originating from anticorrelated noise, photoelectric and Compton EPL images were used as the input to a dictionary-based denoising algorithm. The denoising step showed only minor differences in per-patient calculated aBMD and no differences in the ROC analysis on scout data. This is due to the measurement of a mean value within the vertebral body, averaging out the anticorrelated noise introduced by material decomposition. We expect a beneficial effect of denoising for an automated segmentation algorithm. Noise-suppressed images and raw images lead to the same values in the minimum noise image on a pixel level. This indicates that the noise reduction algorithm does not change quantitative absorption values, as it only removes the anticorrelated noise from spectral maps.

The correlation analysis shows a high degree of correlation (*r* = 0.68) between spectrally measured aBMD with trabecular vBMD. Similar studies comparing DEXA with QCT observed *r* values of 0.61 and between 0.54 and 0.65 [33, 36], suggesting a similar performance of our method to DEXA. With an AUC value of 86% for distinguishing normal from osteopenic plus osteoporotic patients, SDEXA measurements show high classification accuracy with a sensitivity of 84%, and specificity of 83%.

Especially in the low BMD range there is a point cloud with moderate correlation. This can be a result of overestimation of low BMDs in AP views, as the spinal processes overlap, and additional osteophyte formation, vertebral fracture and degenerative changes of the spine can falsify the aBMD value [37–39]. A comparison for the classification accuracy for osteoporosis showed an improved sensitivity and specificity in lateral scout projections. Especially in the low BMD range, the degree of correlation is improved noticeably for 2D and 3D BMD values. Volumetric BMD values were derived by a material decomposition into hydroxyapatite-specific BMD and water and not the reference standard QCT. In recent studies, it was shown that spectrally derived BMD values are on par with conventional QCT measurements [9], or can be even closer to true BMD concentrations [13].

A notable limitation in our patient population is the low number of osteoporotic patients. Only three osteoporotic patients could be analyzed with measured scout data. To impede degraded scout quality, the applied scout dose should be adapted depending on the patient’s body mass index. Furthermore, not all CT protocols feature a scout image including the lumbar spine. An extended field of view on these scout scans leads to increased dose values. However, the additionally applied dose as given in section “Protocol settings” with < 0.06 mSv is very low in comparison to CT applied dose of approximately 6.0 mSv. Especially, a comparison to the current reference standard DEXA is lacking. The reason for this is the retrospective nature of this study. Only a rare minority of patients undergoing abdominal CT examinations received a DEXA scan within a reasonable time interval. A reason for this is the absence of a DEXA device in our radiology department.

In conclusion, we have found a positive correlation between spectral trabecular vBMD and scout scan aBMD (*r* = 0.68) as well as projected AP (*r* = 0.81) and lateral aBMD (*r* = 0.90). A noise reduction technique, as well as an automated mask generation algorithm, was utilized to generate joint BMD values. Using the extended scout scan protocol for opportunistic osteoporosis detection (with an additional dose being only a fraction of the CT dose) could act as a first indicator for a low BMD value and may be earmarked for further investigations.

## Data Availability

The datasets generated and/or analyzed during the current study are not publicly available due to patient protection terms but are available from the corresponding author upon reasonable request.

## Abbreviations

2D: Two-dimensional
3D: Three-dimensional
aBMD: Areal bone mineral density
AP: Anterior-posterior
AUC: Area under the curve
BMD: Bone mineral density
CT: Computed tomography
DEXA: Dual-energy x-tray absorptiometry
EPL: Equivalent path length
QCT: Quantitative CT
ROC: Receiver operating characteristic
ROI: Region of interest
SDEXA: Scout-based DEXA
SNR: Signal-to-noise ratio
vBMD: Volumetric BMD
